# Optic neuritis in an immunocompetent 40-year-old female due to fungal sphenoidal sinusitis

**DOI:** 10.3205/oc000095

**Published:** 2019-03-01

**Authors:** Virna M. Shah, Kartik Panikkar

**Affiliations:** 1 Department of Neuro-Ophthalmology, Aravind Eye Hospital & Postgraduate Institute of Ophthalmology, Coimbatore, Tamilnadu, India; 2 Department of Glaucoma, Aravind Eye Hospital & Postgraduate Institute of Ophthalmology, Coimbatore, Tamilnadu, India

**Keywords:** fungal sphenoidal sinusitis, osteomyelitis, optic neuritis, 6th nerve palsy, immunocompetent

## Abstract

Fungal shpenoidal sinusitis with osteomyelitis is rare in young immunocompetent patients. We present a case of a presumed fungal sphenoidal sinusitis presenting with second and sixth cranial nerve involvement which resolved well with systemic anti-fungal treatment.

## Introduction

Optic neuritis secondary to fungal paranasal sinus diseases is rare and there are very limited reports in literature [1]. Delay in diagnosis and inappropriate treatment with steroids can lead to permanent visual loss [2]. We present a rare case of presumed fungal sphenoidal sinus osteomyelitis presenting with optic neuritis and sixth nerve palsy, which resolved well after systemic anti-fungal drugs.

## Case description

A 40-year-old female patient presented with a painless sudden loss of vision in her right eye of one month duration. The loss of vision was preceded by a binocular diplopia which worsened on the right gaze for the past 3 months. A right sided upper molar tooth extraction was done prior to the onset of symptoms. She also gave a history of repeated headaches for the past 3 years. She had no systemic illnesses. A non-contrast computerized tomography (CT) scan done previously was essentially normal with some non-specific inflammatory changes involving the sphenoid sinus. With a clinical suspicion of right sided optic neuritis, she had received a treatment regimen of injectable steroids for 3 days followed by tapering doses of oral steroids elsewhere.

On examination, her best corrected visual acuity (BCVA) was perception of light in the right eye and 6/6 in the left eye. Anterior segment examination showed a relative afferent pupillary defect in her right eye with an abduction restriction consistent with a right sixth nerve palsy. Fundus examination showed a resolving disc edema in her right eye. Her left eye was normal. Other cranial nerves were normal. A detailed systemic workup including a complete blood count examination showed an elevated erythrocyte sedimentation rate (ESR) of 87 mm/h. Diabetes and any signs of immunosuppression were ruled out. Chest X ray and Mantoux test were negative. 

**Figure 1:**
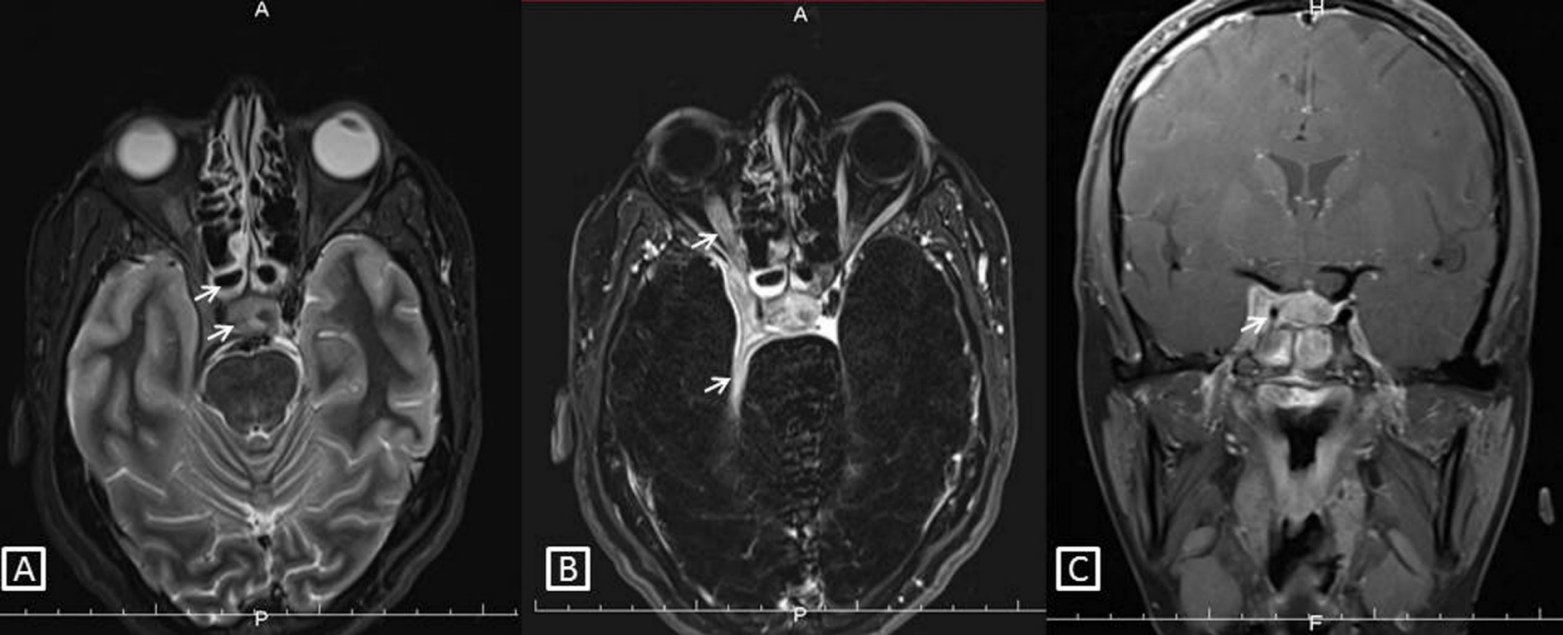
Sphenoidal sinus osteomyelitis. A) Fat suppressed T2 image in the axial plane showing sphenoid sinusitis, altered signal intensity in adjacent bones with expansion of right cavernous sinus by soft tissue (white arrows). B) Subtracted post contrast T1 image in the axial plane showing enhancing soft tissue thickening in the right cavernous sinus with extension to the orbital apex. There is an enhancement of the adjacent right optic nerve and the right half of the optic chiasma shows abnormal signal intensity (white arrows). C) Post contrast T1 image in the coronal plane shows a striking asymmetry of the right cavernous sinus due to enhancing soft tissue within it. Note the narrowed caliber of the cavernous segment of the right internal carotid artery compared to the left (white arrow).

Magnetic resonance imaging (MRI) was advised and mucosal thickening was noted in both compartments of the sphenoid sinus. Areas of altered bone marrow signal intensity with adjacent bone destruction were observed involving the walls of the sphenoid sinus (Figure 1A (Fig. 1)), bilateral posterior clenoid processes, right anterior clenoid process, dorsum sella, the proximal half of clivus, and the right petrous apex region. Posterior one third of the intraorbital portion and of the intracanalicular portion of the right optic nerve, the right half of the optic chiasm was enlarged and showed hyperintense signal intensity on subtracted post contrast T1 image (Figure 1B (Fig. 1)). A hyperintense signal was also noted involving the right optic tract. We also observed a mild enlargement of the pituitary gland and pituitary stalk. Post contrast T1 image in the coronal plane showed an asymmetry of the right cavernous sinus due to enhancing soft tissue within it. (Figure 1C (Fig. 1)). These imaging features were suggestive of fungal sphenoid sinusitis with osteomyelitis involving the right cavernous sinus, adjacent dura, right optic nerve pathway, and pituitary gland.

Opinions of a neurosurgeon and an ophthalmologist were sought and the patient underwent a biopsy of the sphenoidal lesion. Although the biopsy was negative for fungal or bacterial infections, she was empirically started on amphotericin B as clinically and radiologically it was looking like a fungal infection. On follow-up after 1 month, the BCVA in her right eye improved to 3/60, her abduction defect had resolved and the disc was pale, suggesting of a resolved disc edema.

## Discussion

Fungal sinusitis is caused by a wide variety of fungi. Aspergillus is the most common and rhizopus, mucor, cladosporium, candida, and cryptococcus are amongst the others. Isolated sphenoid sinusitis is rare, nevertheless, sphenoid sinusitis occurs in a significant proportion of cases of septic cavernous sinus thrombosis, either in isolation or in combination with involvement of other sinuses [3], [4]. Fungal sinusitis can be associated with infrequent but fatal intracranial extensions. The extension is directed into the skull base either due to proximity of the sinuses or due to pressure necrosis in case of long standing infections. It has to be treated urgently and aggressively as it otherwise invariably proves to be fatal. Therefore, clinical suspicion must be present in cases of refractory sinusitis or long-term sinus disease. The predisposing factors include diabetes mellitus, renal transplant, long-term immunosuppressant medication with steroids, rampant use of antibiotics, poor nutritional status, human immunodeficiency virus infection, and rarely surgery like tooth extraction as seen in our case. The tropical hot and dry climate promotes aspergillus spores and contributes to its wide spread prevalence in the Indian subcontinent [5], [6]. Tuberculous sphenoidal osteomyelitis has also been reported from India [7]. In our case, chest X ray and Mantoux test were negative.

Sphenoid sinusitis produces very few localizing symptoms or external signs. The most common symptom is headache, which can be retrobulbar, parietal or frontal. Fever occurs in over one half of the patients with acute sphenoid sinusitis. The sphenoidal sinuses bear a close anatomical relationship with the cranial nerves II to IV, the dura mater, pituitary gland, cavernous sinus, internal carotid artery, sphenopalatine artery, and pterygopalatine nerve which have been reported to be infected by dissemination [8]. In our case, second and sixth cranial nerves were involved. 

MRI is superior to CT scan for early diagnosis of skull base osteomyelitis. On injection of contrast, enhancement is seen. T2 hypo intense material within sinus cavity, bony involvement, interruption of enhancing mucosa with continuous inflammation to adjacent structures are the salient features [9] which were seen in our case. Diagnosis can be confirmed by tissue sampling using CT-guided fine-needle aspiration. In this series, elevated white blood cell count, fever, and abnormal blood cultures were absent. An elevated ESR level raised concern for skull base osteomyelitis. This blood panel was very similar to our patient’s report. 

The mainstay of medical treatment is antifungal therapy with amphotericin B. It is generally used in patients having creatinine >2.5 mg. The required dosage is 4 mg/kg/day and could be increased up to 10–15 mg/kg/day [10].

In conclusion, intracranial extension of fungal sinusitis should be treated as a medical emergency, as the outcome is fatal in most of the cases. For early diagnosis, a high degree of clinical suspicion is required. The presence of a highly invasive fungal osteomyelitis in an immunocompetent patient with no other predisposing factors was brought forth by a high degree of clinical suspicion seconded by adequate radiological evidence.

## Notes

### Competing interests

The authors declare that they have no competing interests.
